# Prevalence, determinants and co-morbidities of chronic kidney disease among First Nations adults with diabetes: results from the CIRCLE study

**DOI:** 10.1186/1471-2369-13-57

**Published:** 2012-07-09

**Authors:** Roland F Dyck, Mariam Naqshbandi Hayward, Stewart B Harris

**Affiliations:** 1 Department of Medicine, Royal University Hospital, 103 Hospital Drive, Saskatoon, SK, S7N 0W8, Canada; 2 Department of Community Health and Epidemiology, University of Saskatchewan, Saskatoon, SK, S7N 5E5, Canada; 3 Centre for Studies in Family Medicine, Department of Family Medicine, The University of Western Ontario, London, Ontario, N6G 4X8, Canada

**Keywords:** Indigenous peoples, Aboriginal, First Nations, Diabetes, Chronic kidney disease, End stage renal disease, Risk factors.

## Abstract

**Background:**

Indigenous peoples worldwide are experiencing elevated rates of type 2 diabetes and its complications. To better understand the disproportionate burden of diabetic end stage renal disease (ESRD) among Canadian First Nations people (FN), we examined prevalence, determinants, and co-morbidities of chronic kidney disease (CKD) within this population.

**Methods:**

The 2007 Canadian FN Diabetes Clinical Management and Epidemiologic (CIRCLE) study conducted a cross-sectional national medical chart audit of 885 FN adults with type 2 diabetes to assess quality of diabetes care. In this sub-study, participants were divided by estimated glomerular filtration rate (eGFR in ml/min/1.73 m^2^), as well as by albuminuria level in those with eGFRs = > 60. Those with eGFRs = > 60 and negative albuminuria were considered to have normal/near normal kidney function (non-CKD). Using univariate and logistic regression analysis, they were compared with participants having eGFRs = > 60 plus albuminuria (CKD-alb) and with participants having eGFRs <60 (CKD-eGFR <60).

**Results:**

While 84.5% of total CIRCLE participants had eGFRs = > 60, almost 60% of the latter had CKD-alb. Of the 15.5% of total participants with CKD-eGFR <60, 80% had eGFRs 30–60 (Stage 3 CKD) but over 10% (1.6% of total participants) had ESRD. Independent determinants of CKD-alb were male gender and increasing diabetes duration, systolic BP, A1C and total cholesterol. These plus smoking rates also discriminated between FN with micro- and macro-albuminuria. Independent determinants of CKD-eGFR <60 were increasing age at diabetes diagnosis, diabetes duration, total cholesterol and systolic BP. However, participants with CKD-eGFR <60 also displayed a decreasing mean age of diabetes diagnosis as eGFR declined. Micro-vascular co-morbidities were significantly associated with CKD-alb but both micro- and macro-vascular co-morbidities were associated with CKD-eGFR <60. Only 35-40% of participants with CKD used insulin.

**Conclusions:**

High prevalences of CKD-alb and early CKD-eGFR <60 among diabetic FN were largely related to modifiable and treatable risk factors. However, an earlier age of diabetes diagnosis and longer duration of diabetes characterized those with ESRD. These findings suggest that a failure to meet current standards of diabetes care interacting with an age-related survival benefit contribute to the disproportionate burden of ESRD among FN and possibly other Indigenous peoples.

## Background

Rates of diabetic end stage renal disease (ESRD) are disproportionately elevated among Indigenous peoples worldwide [1,2]. In Canada, the incidence of diabetic ESRD remains three times higher among First Nations people (FN) than others, although disparities have diminished slightly since peaking in the early 1990s [3]. After adjusting for substantially higher diabetes prevalence among FN compared to non-FN [4], these disparities are characterized by two important observations. First, compared with their non-FN counterparts, a greater proportion of diabetic FN have normal estimated glomerular filtration rates (eGFRs) but a higher prevalence of albuminuria [5-8]. Second, FN are younger at diabetes diagnosis than non-FN and experience a longer duration between diabetes and ESRD diagnoses [3]. These findings suggest that diabetic FN are more prone to the initial development of chronic kidney disease (CKD) and may be more likely to survive long enough to develop ESRD [6,9].

Although the analysis of administrative [3,4,8] and other large databases [10,11] has greatly enhanced our understanding of the differences between FN and non-FN in the epidemiology of diabetes and ESRD, the possible genetic and environmental factors that underlie the observations summarized above remain unclear. Because the current investigation utilized detailed individual level information gathered as part of the Canadian First Nations Diabetes Clinical Management and Epidemiologic (CIRCLE) study [12], we were able to conduct the first comprehensive study of prevalence, determinants, treatment and co-morbidities of chronic kidney disease within a broad sample of adult FN people with type 2 diabetes across Canada. The results provide new insights into the development and progression of diabetic renal disease within this population which we hope can be translated into more effective prevention/management initiatives.

## Methods

### Research Design, Ethics Approval and Participant Selection

The methodology used in the CIRCLE study has been previously described [12]. Briefly, we carried out a cross-sectional medical chart audit of 2007 data for 885 FN adults (18 years and older) with type 2 diabetes who lived in 19 First Nations communities across seven provinces in Canada. FN are one of three diverse Indigenous populations of Canada which also include Inuit and Métis. The study was approved by the University of Western Ontario, the University of Toronto and Health Canada Research Ethics Boards as well as by each Band Council and other University ethics review boards as required. Community participation was confirmed by a research agreement after community consultations were held. The Ownership, Control, Access and Possession (OCAP) principles developed by the National Aboriginal Health Organization [13] were used to guide the ethical conduct of the study.

A registry of all adults with type 2 diabetes was developed in each community. Potential CIRCLE participants were then randomly selected from each community’s registry and sequentially contacted for consent until up to 50 people had agreed to participate. For the final analyses, medical chart auditing was successfully carried out by trained local research assistants on 885 of 950 potential study participants.

### Data Collection and Definition of Chronic Kidney Disease Sub-Groups

Charts were audited for participant characteristics, a range of diabetes quality care indicators derived from clinical practice guidelines (CPG) [14], pharmacologic treatment and the presence of co-morbidities that are defined and described in more detail elsewhere (12). Audits were carried out in 2008/09 for the latest healthcare information available for 2007 which was based on a mean of 8.7 visits per participant. If data was missing for 2007, the most recent value prior to that year was used. All information was de-identified and entered into an Access database using study identification numbers.

CIRCLE participants were sub-divided into groups based on their estimated glomerular filtration rates (eGFR) and urinary excretion of albumin. Because test results were obtained from medical chart records, methods for measuring serum creatinine and urine parameters were those being used by the particular province rather than being carried out in one centralized laboratory. eGFRs were calculated using an equation based on the original Modification of Diet in Renal Disease Study Group (MDRD) equation which uses age, sex, serum creatinine, and black/white ethnicity [15]. Thus for males: eGFR = 186 x (serum creatinine x 0.0113)^-1.154^ x age (years)^-0.203^ and for females: eGFR = 186 x (serum creatinine x 0.0113)^-1.154^ x age (years)^-0.203^ x 0.742. For ethnicity, we used the unadjusted (white ethnicity) parameter which has been preliminarily validated in a North American Indian population [16]. Only those who had at least one serum creatinine result during the study year were included in this sub-study. When more than one result was available, we utilized the lowest value to avoid results possibly associated with acute renal impairment.

The eGFR sub-groups were based on chronic kidney disease (CKD) stages [17] using the Kidney Disease Outcomes Quality Initiatives classification (eGFR = > 90 ml/min per 1.73 m^2^ = no CKD and stage 1, 60–89.9 = no CKD and stage 2, 30–59.9 = stage 3, 15–29.9 = stage 4, <15 = stage 5). Those with an eGFR <15 who were not receiving renal replacement therapy (stage 5a) were combined with stage 4 because of small numbers, while people receiving renal replacement therapy were classified as stage 5b. Finally, because there are particular limitations to the MDRD eGFR equation at eGFRs = > 60ml/min/1.73m^2^ (see DISCUSSION), we presented both overall data for those with eGFRs = > 60ml/min/1.73m^2^ as well as sub-group information for those with eGFRs = > 90 and 60–90ml/min. Those with an eGFR = > 60 were further sub-divided by the presence or absence of albuminuria [14]. Micro-albuminuria was defined as a 24h urine value 30–300mg/day for both sexes, or a urine albumin: creatinine ratio (ACR) of 2.0-20.0mg/mmol for males, or an ACR of 2.8-28mg/mmol for females. Macro-albuminuria was defined as a 24h urine value >300mg/day for both sexes, an ACR >20.0 mg/mmol for males or an ACR >28 mg/mmol for females. Ten individuals without an available ACR record were considered to have micro-albuminuria because of a chart diagnosis by a physician.

### Statistical Analyses

After determining the distribution of CIRCLE participants by eGFR stage (all) and by albuminuria positivity (those with eGFRs = > 60ml/min), we defined three groups for further comparisons. Those with normal or near-normal kidney function (non-CKD) had eGFRs = > 60ml/min/1.73m^2^ and negative albuminuria. CKD was defined on the basis of positive albuminuria in those with eGFRs = > 60ml/min/1.73m^2^ (CKD-alb) or an eGFR <60ml/min/1.73m^2^ regardless of albuminuria (CKD-eGFR <60). All subsequent comparisons were between people with either CKD-alb or CKD-eGFR <60 and those with non-CKD; we compared overall groups as well as subgroups defined by eGFR stage.

The following parameters were compared between groups and sub-groups: gender; mean age at audit and diabetes diagnosis, and duration of diabetes; mean body mass index (BMI); proportion of smokers; mean systolic blood pressure (sBP); mean diastolic blood pressure (dBP); mean glycosylated haemoglobin (A1C), mean total cholesterol (TC); mean low density lipoprotein cholesterol (LDL); mean high density lipoprotein cholesterol (HDL); proportions with micro-vascular and macro-vascular co-morbidities (total and by organ system); and proportions receiving treatment with insulin and/or agents that target the renin/angiotensin/aldosterone system (RAAS).

Univariate analyses employed chi square tests to compare proportions and two tailed t-tests to compare means. We also used trend analysis to compare selected parameters by level of albuminuria (negative, micro-albuminuria, macro-albuminuria) in those with eGFRs = > 60ml/min. Finally, multivariate analysis was carried out by entering potential determinants of CKD-alb or CKD-eGFR <60 (versus non-CKD) into a logistic regression model. Statistical tests were conducted using PASW Statistics version 18.0 and p <0.05 was used to determine significance. Odds ratios (OR) and 95% confidence intervals (CI) were determined in multivariate analyses.

## Results

Of 885 CIRCLE participants, 850 had at least one serum creatinine measured during the audit year of 2007. Table 1 shows the proportions of those individuals by eGFR stage. Urine albumin measurement was carried out in 685 of the 850 participants but the eGFR distribution of persons with and without a urine albumin test was almost identical. A large majority of total study participants (84.5%) had an eGFR ≥60 ml/min. Of the latter who also had a urine albumin test (583), 242 (41.5%) had negative albuminuria (non-CKD) and 341 (58.5%) had albuminuria (CKD-alb). Only 132 (15.5%) of total study participants had an eGFR <60 ml/min/1.73 m^2^ (CKD-eGFR <60) including 8 (0.9%) who were receiving dialysis. People with CKD-alb, CKD-eGFR <60 and non-CKD totalled 715 people and were the subjects of further analyses. Their mean age at time of audit was 55.6 years, and 65.3% were female.

**Table 1:** Circle participants by eGFR* category and urine albumin testing

**Participant Numbers**	**Total**	**eGFR = > 90**	**eGFR 60 to <90**	**eGFR 30 to <60**	**eGFR 15 to <30**	eGFR <15 (no RRT**)	**eGFR <15 (RRT)**
		**Non/Stage 1 CKD**	**Non/Stage 2 CKD**	**Stage 3 CKD**	**Stage 4 CKD**	**Stage 5a CKD**	**Stage 5b CKD**
People with eGFR (% total)	850	451 (53.1)	267 (31.4)	**105** (12.4)	**13** (1.5)	**6** (0.7)	**8** (0.9)
People with eGFR & Urine Albumin Test (% total)	685	**367** (53.5)	**216** (31.5)	84 (12.3)	10 (1.5)	4 (0.6)	4 (0.6)
Normal Urine Albumin (% in eGFR Stage)	274	147 (40.1)	95 (44.0)	30 (35.7)	1 (10)	1 (25)	0 (0)
Micro-albuminuria (% in eGFR Stage)	278	159 (43.3)	88 (40.7)	29 (34.5)	2 (20)	0 (0)	0 (0)
Macro-albuminuria (% in eGFR Stage)	133	61 (16.6)	33 (15.3)	25 (29.8)	7 (70)	3 (75)	4 (100)
Micro/Macro-albuminuria (% in eGFR Stage)	411	220 (59.9)	121 (56.0)	54 (64.3)	9 (90)	3 (75)	4 (100)
People included in further analyses (% total)***	**715**	**367** (51.3)	**216** (30.2)	**105** (14.7)	**13** (1.8)	**6** (0.8)	**8** (1.1)

*Estimated glomerular filtration rates in ml/min/1.73 m^2^ – categories correspond to Chronic Kidney Disease (CKD) Stages. Those with eGFRs = > 60 and no albuminuria are non-CKD. Stage 1 plus Stage 2 = CKD-alb. Stages 3-5b = CKD-eGFR <60.

**Renal replacement therapy.

***Those with eGFR = > 60 and a urine albumin test plus those with eGFR <60 with or without urine albumin test (Numbers in **bold.**)

Table 2 - Characteristics and comparison of non-CKD and CKD-alb participants: Despite only 34.7% of males in the total study group, people with CKD-alb were more likely to be male than those with non-CKD (39.0% versus 30.2% respectively; p = 0.017). CKD-alb participants also had a significantly longer duration of diabetes (10.3 versus 7.8 years; p < 0.0001) despite being similar in age at time of diabetes diagnosis. Most people were obese (mean BMI 33.8). There was not a significant difference in adiposity between CKD-alb and non-CKD participants although there was a trend for the former to have lower BMIs. Similarly, smoking rates were high overall (38.7%) but did not differ significantly by albuminuria positivity.

**Table 2:** Characteristics of Circle participants by eGFR category and urine albumin (Ua) positivity (+/−) when eGFR = > 60

PARAMETER* (Mean values or %)	**eGFR Groups**	**eGFR = < 90**			**eGFR 60 - < 90**			eGFR = > 60 Total**			**eGFR 30 - <60**		**eGFR <30 (no RRT)**		**eGFR <15 RRT**		**All eGFR < 60 = CKD-eGFR <60**	
	**Total**	**Non-CKD (UA-)**	**CKD-alb (UA+)**	**p**	**Non-CKD (UA-)**	**CKD-alb (UA+)**	**p**	**Non-CKD (UA-)**	**CKD-alb (UA+)**	**p**	**Stage 3 CKD**	**p vs Non-CKD**	**Stage 4/5a CKD**	**p vs Non- CKD**	**Stage 5b CKD**	**p vs Non- CKD**	**Stage 3-5b CKD**	**p vs Non- CKD**
NUMBER	715	147	220	…	95	121	…	242	341	…	105	…	19	…	8	…	132	…
% FEMALE	65.3	68.7	63.6	n/s	71.6	56.2		69.8	61.0	0.017	72.4	n/s	68.4	n/s	12.5	0.002	68.2	n/s
AGE AT AUDIT	55.6	47.6	49.4	n/s	59.6	61.3	n/s	52.3	53.6	n/s	68.1	0.000	62.7	0.001	56.0	n/s	66.6	0.000
age diagnosis	44.3	39.7	39.4	n/s	49.9	48.1	n/s	43.6	42.4	n/s	53.0	0.000	42.2	n/s	35.9	n/s	50.4	0.000
duration diabetes	10.4	7.1	9.3	0.001	8.8	12.3	0.001	7.8	10.3	0.000	14.3	0.000	20.1	0.000	19.1	0.000	15.4	0.000
BMI	33.8	35.6	33.4	n/s	34.8	33.7	n/s	34.7	33.5	n/s	33.3	n/s	33.6	n/s	27.5	0.004	32.9	0.033
% SMOKER	38.7	50	48.5	n/s	30.4	27.3	n/s	42.2	41.1	n/s	23	0.001	27.8	n/s	37.5	n/s	26.2	0.002
BLD PRESSURE																		
sBP (mm Hg)	132.2	128.2	132.3	0.016	129.4	135.1	0.015	128.7	133.3	0.001	135.6	0.002	137.5	0.018	139.3	n/s	136.1	0.000
dBP (mm Hg)	75.5	76.3	77.3	n/s	75.4	74.9	n/s	75.9	76.4	n/s	72.2	0.002	74.05	n/s	72.8	n/s	72.5	0.002
BLOOD TESTS																		
A1C (mean %)	8.14	8.05	8.95	0.000	7.18	8.19	0.000	7.72	8.68	0.000	7.57	n/s	7.65	n/s	6.25	0.033	7.5	n/s
TC (mM)	4.52	4.38	4.70	0.006	4.37	4.45	n/s	4.38	4.61	0.008	4.48	n/s	4.84	n/s	4.66	n/s	4.54	n/s
LDL (mM)	2.38	2.34	2.44	n/s	2.33	2.33	n/s	2.33	2.40	n/s	2.39	n/s	2.6	n/s	2.44	n/s	2.43	n/s
HDL (mM)	1.21	1.21	1.25	n/s	1.19	1.18	n/s	1.20	1.22	n/s	1.23	n/s	1.23	n/s	1.04	n/s	1.22	n/s
COMORBIDITIES																		
All Macrovasc (%)	21.1	11.6	10.5	n/s	23.2	25.6	n/s	16.1	15.8	n/s	38.1	0.000	68.4	0.000	62.50	0.005	43.9	0.000
% periph vasc	3.9	0.7	1.4	n/s	3.2	5.0	n/s	1.7	2.6	n/s	4.8	n/s	31.6	0.000	50.0	0.000	11.4	0.000
% cerebro vasc	5.3	2.7	2.7	n/s	4.2	5.8	n/s	3.3	3.8	n/s	12.4	0.002	15.8	0.037	12.5	n/s	12.9	0.001
% combined heart	16.1	9.5	7.3	n/s	17.9	19.8	n/s	12.8	11.7	n/s	29.5	0.000	52.6	0.000	37.5	n/s	33.3	0.000
All Microvasc (%)	22.4	11.6	23.2	0.003	11.6	25.6	0.007	11.6	24.0	0.000	32.4	0.000	47.4	0.000	87.5	0.000	37.9	0.000
% foot disease	4.6	2.0	5.0	n/s	0.0	2.5	n/s	1.2	4.1	0.034	8.6	0.002	21.1	0.001	37.5	0.000	12.1	0.000
% eye disease	13.8	6.1	13.6	0.015	9.5	15.7	n/s	7.4	14.4	0.006	20.0	0.001	21.1	0.063	85.5	0.000	24.2	0.000
% neuropathy	12.7	6.1	13.2	0.021	7.4	14.0	n/s	6.6	13.5	0.005	17.1	0.003	21.1	0.046	87.5	0.000	22.0	0.000
MEDICATIONS																		
% Insulin	30.1	18.4	33.2	0.001	21.1	38	0.005	19.4	34.9	0.000	30.5	0.018	57.9	0.000	75	0.001	37.1	0.000
% RAAS agents	75.2	63.3	76.8	0.004	73.7	85.1	0.028	67.4	79.8	0.001	81.9	0.004	63.2	n/s	62.5	n/s	78.0	0.019

* BMI-body mass index, sBP-systolic blood pressure, dBP-diastolic Blood pressure, A1C-glycosylated hemoglobin, TC-total cholesterol, LDL-LDL cholesterol, HDL-HDL cholesterol; periph vasc-peripheral vascular disease; cerebro vasc-cerebral vascular disease; combined heart- coronary artery disease and/or left ventricle hypertrophy and/or congestive heart failure. RAAS agents- containing angiotensin converting enzyme inhibitors or angiotensin receptor blockers.

**Total non-CKD is comparison group for all sub-groups and total with eGFR < 60.

Mean sBP was higher among those with CKD-alb than non-CKD (133.3 versus 128.7 mm Hg; p < 0.001) but mean dBP was similar. Mean A1C was almost one percentage higher in those with CKD-alb compared to those with non-CKD (8.68 versus 7.72; p < 0.0001), while the mean TC was the only lipid value that was significantly higher among those with CKD-alb. Microvascular but not macrovascular complications were significant co-morbidities associated with CKD-alb. Finally, almost 35% of CKD-alb participants were receiving insulin therapy and almost 80% were on RAAS agents. These proportions were significantly higher than those for non-CKD.

With respect to participants having an eGFR = > 60 ml/min, those with an eGFR = > 90 were younger at time of diabetes diagnosis, and had a shorter duration of diabetes than those with an eGFR from 60 to <90 ml/min. Those with a higher eGFR also had higher A1C values; the worst A1C levels were in those with eGFRs = > 90 ml/min/1.73 m^2^ and positive albuminuria (8.95 versus 8.05 in those with no albuminuria; p < 0.0001).

Table 2 – Characteristics of participants with CKD-eGFR ≤60 and comparison with non-CKD participants: There continued to be a female predominance overall but 7 of 8 people on dialysis were male. While the mean age at diabetes diagnosis for people with an eGFR 30 to <60 ml/min/1.73 m^2^ (stage 3 CKD) was almost 10 years older than non-CKD participants (52.95 versus 43.62 years; p < 0.0001), the mean age at diabetes diagnosis of people with eGFRs <30 ml/min/1.73 m^2^ was younger (42.17 for those in Stage 4/5a and 35.88 for those in Stage 5b). This was associated with a longer duration of about 20 years from diabetes diagnosis in the latter two groups compared to 14.3 years for those with stage 3 CKD.

Participants with CKD-eGFR <60 were slightly less obese and had significantly lower smoking rates than those with non-CKD. Mean sBP was significantly higher but mean dBP was significantly lower compared to non-CKD. There were no overall differences in A1C or lipid values between people with CKD-eGFR <60 and non-CKD; however, the mean A1C level was significantly lower among those on dialysis (6.25 versus 7.72; p = 0.033). In contrast to participants with CKD-alb, those with CKD-eGFR <60 not only experienced a higher frequency of microvascular but also of macrovascular co-morbidities.

Table 3 shows the characteristics of CIRCLE participants with eGFR = > 60 ml/min/1.73 m^2^ by level of albuminuria (none, micro-albuminuria, macro-albuminuria). There was a progressive increase in the proportion of males with increasing albuminuria from 30.2% of those with no albuminuria to 50% of those with macro-albuminuria (p = 0.003). Those with macro-albuminuria were also significantly younger at time of diabetes diagnosis (38.5 years versus almost 44 years in other two groups; p = 0.002), and had a longer duration of diabetes (11.5 years versus 9.9 years and 7.8 years for those with micro-albumiuria and no albuminuria respectively; p < 0.0001). People with macro-albuminuria were significantly less obese (mean BMI 31.6 versus >34 in the two other groups; p = 0.001), and were more likely to smoke (52.2% versus 36.7% and 42.2% of those with micro-albuminuria and no albuminuria respectively; p = 0.04).

**Table 3:** Characteristics of Circle participants with eGFR = > 60 by degree of albuminuria

PARAMETER*	**People With eGFR = > 60 and Urine Albumin (UA) Test**				
	**Total With UA**	**UA neg (non-CKD)**	Micro-albuminuria**	Macro-albuminuria**	**p**
NUMBER (%)	583 (100)	242 (41.5)	247 (42.4)	94 (16.1)	…
% FEMALE	64.7	69.8	65.2	50.0	0.003
AGE AT AUDIT – mean (SD)	53.1 (13.8)	52.3 (13.6)	54.6 (14.0)	50.9 (13.5)	0.046
at diabetes diagnosis – mean (SD)	42.9 (13.1)	43.6 (12.8)	43.9 (132.1)	38.5 (13.2)	0.002
diabetes duration – mean (SD)	9.3 (6.7)	7.8 (5.9)	9.9 (7.0)	11.5 (7.2)	0.000
BODY MASS INDEX – mean (SD)	34.0 (6.8)	34.70 (6.4)	34.33 (7.4)	31.59 (5.7)	0.001
% SMOKER	41.6	42.2	36.7	52.2	0.04
BLOOD PRESSURE					
sBP mm Hg – mean (SD)	131.4 (16.8)	128.7 (15.1)	133.5 (17.4)	132.7 (18.5)	0.004
dBP mm Hg – mean (SD)	76.2 (10.7)	75.9 (10.2)	77.0 (10.5)	75.11 (12.3)	n/s
BLOOD TESTS					
AIC % – mean (SD)	8.28 (2.26)	7.72 (1.92)	8.34 (2.21)	9.58 (2.64)	0.000
TC mM – mean (SD)	4.51 (1.04)	4.38 (0.93)	4.49 (1.01)	4.93 (1.24)	0.000
LDL mM – mean (SD)	2.37 (1.82)	2.33 (0.77)	2.31 (0.81)	2.65 (0.93)	0.003
HDL mM – mean (SD)	1.22 (0.30)	1.20 (0.29)	1.20 (0.30)	1.30 (0.33)	0.017
COMORBIDITIES					
% eye disease	11.5	7.4	13	18.1	0.015
% neuropathy	10.6	6.6	11.7	18.1	0.007
MEDICATIONS					
% Insulin	28.5	19.4	33.2	39.4	0.000
% RAAS agents	74.6	67.4	75.7	90.4	0.000

*sBP-systolic blood pressure, dBP-diastolic blood pressure, A1C-glycosylated hemoglobin, TC-total cholesterol, LDL-LDL cholesterol, HDL-HDL cholesterol, RAAS agents- containing angiotensin converting enzyme inhibitors or angiotensin receptor blockers.

**Micro-albuminuria plus Macro-albuminuria. = CKD-alb.

People with any level of albuminuria had a significantly higher mean sBP (>130 mm Hg) than those with non-CKD (128.7 mm Hg) but mean dBP’s were similar and normal (<80 mm Hg) in all groups. There was a progressive and highly significant increase in mean A1C by increasing amounts of albuminuria, from 7.72% in those with no albuminuria to 8.34% and 9.58% in those with micro- and macro-albuminuria respectively (<0.0001). TC also increased progressively from 4.38 mmol/L in those with non-CKD to 4.49 and 4.93 mmol/L in those with micro- and macro-albuminuria respectively (<0.0001). Participants with macro-albuminuria also had significantly higher levels of LDL and HDL cholesterol.

The proportion of CIRCLE participants with eye disease and neuropathy increased progressively and significantly by level of albuminuria from under 8% in those with no albuminuria to over 18% in those with macro-albuminuria. Finally, although use of both insulin and RAAS agents increased significantly with degree of albuminuria, only 39.4% of those with macro-albuminuria were on insulin while over 90% of that group were taking RAAS agents.

Table 4 shows the results of multivariate analyses comparing CKD-alb and CKD-eGFR <60 participants with those having non-CKD. Using logistic regression, male gender (OR 1.86; 95% CI’s 1.19, 2.90), diabetes duration (OR 1.04 per year; 95% CI’s 1.01, 1.08), increasing sBP (OR 1.02 per mm Hg; 95% CI’s 1.003, 1.03), increasing A1C (OR 1.19 per 0.1%; 95% CI’s 1.07, 1.33) and increasing TC (OR 1.32 per 0.1 mM; 95% CI’s 1.06, 1.64) were all independent determinants for CKD-alb. While increasing age at diabetes diagnosis (OR 1.06 per year; 95% CI’s 1.03, 1.09), diabetes duration (OR 1.19 per year; 95% CI’s 1.13, 1.26) and increasing TC (OR 1.47 per 0.1 mM; 95% CI’s 1.08, 2.01) were also independent determinants for CKD-eGFR <60, sBP almost achieved significance as well (OR 1.02 per mm Hg; 95% CI’s 0.999, 1.04; p = 0.059).

**Table 4:** Adjusted odds ratios (OR) for associations between model variables and chronic kidney disease*

**VARIABLE**	**People With eGFR ≥60 and Albuminuria (CKD-alb) versus Non-CKD**			**People With eGFR <60 (CKD-eGFR <60) versus Non-CKD**		
	**OR**	95% CI**	**sig**	**OR**	**95% CI**	**sig**
Gender (male versus female)	1.86	1.19, 2.90	0.006	1.86	0.91, 3.78	0.088
Diabetes Diagnosis Age (per year ↑)	0.9	0.97, 1.01	0.366	1.06	1.03, 1.09	<0.0001
Diabetes Duration (per year ↑)	1.04	1.01, 1.08	0.023	1.19	1.13, 1.26	<0.0001
Body Mass Index (per 1 unit ↑)	0.99	0.96, 1.02	0.450	1.00	1.00, 1.00	0.552
Smoker (yes versus no)	1.01	0.66, 1.55	0.979	0.97	0.47, 1.97	0.923
Systolic BP (per 1 mmHg ↑)	1.02	1.003, 1.03	0.019	1.02	0.999, 1.04	0.059
Diastolic BP (per 1 mmHg ↑)	0.99	0.97, 1.02	0.99	0.99	0.95, 1.02	0.424
A1C (per 0.1% ↑)	1.19	1.07, 1.33	0.002	1.00	0.82, 1.21	0.96
Total Cholesterol (per 0.1 mM ↑)	1.32	1.06, 1.64	0.014	1.47	1.08, 2.01	0.015

*Chronic Kidney Disease = eGFR ≥60 with albuminuria (CKD-alb) or eGFR <60 with/without albuminuria (CKD-eGFR <60).

**CI-confidence interval.

## Discussion

While recent reports have examined the relationship between chronic kidney disease (CKD) and diabetes in other North American indigenous populations [18-20], this is the most comprehensive study of prevalence, determinants and co-morbidities of chronic kidney disease (CKD) that has been carried out among adult First Nations people (FN) with type 2 diabetes in Canada. It is also the first to include a broad representation of FN communities from across the country. We have confirmed that a large majority of diabetic FN have normal or near normal eGFRs but experience high rates of albuminuria [5-8], and that those who develop ESRD are characterized by a younger age at diabetes diagnosis and a longer exposure to the metabolic consequences of diabetes [3,9]. What we now show more clearly than previously reported is that the initial development of CKD in this population is strongly related to modifiable and treatable risk factors despite possible genetic and prenatal precursors. Furthermore, the development of ESRD appears dependent on factors contributing to long term survival of a small minority rather than to rapid progression of CKD.

Almost 60% of diabetic FN with eGFRs = > 60 ml/min/1.73 m^2^ in our study had micro- or macro-albuminuria (CKD-alb) and this was clearly associated with suboptimal diabetes management. Thus, we not only demonstrated for the first time in this population that increasing A1C, sBP, and TC were all independent determinants of CKD-alb but that progressive increases in A1C and TC also discriminated between those with micro- and macro-albuminuria, with the latter group having mean A1C’s >9.5%. Additionally, over 50% of those with macro-albuminuria were smokers, more than 10 percentage points higher than those with micro-albuminuria or non-CKD. It is thus concerning to note that smoking rates were also very high among the youngest sub-group in our study, those with eGFRs 90 and higher. Interestingly, increasing BMI was not associated with CKD-alb and those with macro-albuminuria had lower BMI’s than others. This might be due to weight loss associated with very poor glycemic control and/or, because of their inadequate usage, lack of the weight gain often related to glucose lowering drugs.

A longer duration of diabetes and male gender were also independent determinants of albuminuria. The impact of disease duration was expected but has not been previously shown in this population. Similarly, while a higher frequency of albuminuria has been reported among male FN with diabetes [6], this is the first time that male gender has been identified as an independent determinant of albuminuria among FN. Those with macro-albuminuria, which included the highest proportion of males, also had the highest A1C, TC and smoking rates. Therefore, it is likely that differences in lifestyle and diabetes management rather than underlying biologic differences largely account for the impact of gender on development of albuminuria.

This study also provides insights into why diabetic FN experience a higher risk for ESRD. Although independent determinants of an eGFR <60 ml/min/1.73 m^2^ included increases in age at diabetes diagnosis, duration of diabetes, sBP and TC, we found that the mean age at diabetes diagnosis actually decreased from 53.0 years for those with Stage 3 CKD to 35.9 years for those with Stage 5b CKD. Furthermore, there was a reversal in the female: male ratio from predominantly females in Stage 3 to overwhelmingly males in Stage 5b. These are consistent with Saskatchewan findings [6] and are most obviously explained by an age-related survival effect whereby those younger at diabetes diagnosis were more likely to survive after reaching Stage 3 CKD. Unlike overall mortality rates among diabetic FN which tend to be worse for males [21], our findings from this and previous studies [3,6] suggest that mortality during the late stages of diabetic CKD appears to be higher for females. Importantly, we have now provided evidence that differential mortality likely contributes to both ethnicity-based disparities in development of diabetic ESRD [6] as well as to ESRD risk within FN populations.

As we and others have reported [6,22], mean A1C levels actually decreased with declining eGFRs and were lowest (6.25%) in those requiring RRT. This observation may be due to uremia-related appetite suppression and nutritional problems as supported by a parallel decline in BMI, or to a reduction in exogenous insulin requirements related to declining renal function. Since it has recently been shown that A1C values <6.5% are associated with increased mortality among those with diabetic CKD [19], this may be a factor in the poor median survival of both FN and non-FN with diabetic ESRD [3].

While only micro-vascular comorbidities were significantly associated with CKD-alb, both micro- and macro-vascular comorbidities were related to CKD-eGFR <60. The first finding is presumably due to the impact of poor glycemic control on the development and progression of micro-vascular complications [23,24]. The second finding is also important because the association of macro-vascular co-morbidities with worsening eGFR relates to both the underlying pathophysiology of these complications and the implications for those with eGFRs <60 ml/min. Thus, increasing TC, sBP and diabetes duration were all independent determinants of CKD-eGFR <60, but are also known risk factors for macro-vascular complications and death in those with diabetes [25,26]. Furthermore, impaired renal function also contributes to the development of macro-vascular complications and death [27,28]. Accordingly, our findings not only provide a plausible explanation for mechanisms contributing to the development of CKD-eGFR <60 and its co-morbidities, but also for the attendant increase in mortality rates. Combined with the higher AIC levels that we identified in those with earlier stages of CKD and that have also been shown to be independently associated with death in those with CKD [22], it is not surprising that only a select minority of people with diabetes live long enough to develop ESRD and that this survivorship particularly favours those with a younger age of diabetes onset.

Although people with CKD-alb and CKD-eGFR <60 were more likely to be on insulin and RAAS agents than people with non-CKD, only the 80% on RAAS agents approached Canadian Diabetes Association CPG guidelines recommending their use in those with CKD [14]. In contrast, only about 1/3 of FN with CKD were on insulin – in the context of a mean A1C >8% for the total study population and over 9.5% in those with macro-albuminuria, these results indicate a critical need for improvements in glycemia management. This is particularly concerning when guidelines recommend insulin therapy in those with A1Cs consistently >9% [14] and when we have previously shown that diabetic FN are less likely than their non-FN counterparts to achieve a target A1C of 7% [29]. Finally, while we did not analyze the use of lipid lowering agents in this sub-study, over 70% of CIRCLE participants with an LDL >2.5% were receiving pharmacologic treatment. As we have noted previously [29], LDL values tend to be closer to target than other diabetes quality care indicators among FN and may not adequately reflect dyslipidemia in this population. Instead, TC was an independent determinant of both CKD-alb and CKD-eGFR <60.

Strengths of this study included its national scope, randomized approach to participant selection, ability to only include those with type 2 diabetes and use of detailed individual level information. Limitations included the use of opportunistic rather than random sampling of FN communities across Canada. Thus, study results do not necessarily represent the findings of all FN people with diabetes in Canada. For example, we observed a smaller proportion of CIRCLE participants requiring RRT compared to their Saskatchewan FN counterparts [6], possibly because of difficulty contacting such individuals who frequently travel to or have relocated to larger centers where dialysis facilities are available. Second, we were unable to identify CKD with causes other than diabetes and did not have complete data with respect to urine albumin testing. Third, in some cases, a diagnosis of CKD-alb or CKD-eGFR <60 may have been made on the basis of a single urine albumin or serum creatinine test result and we can not rule out the possibility that some abnormal results were due to acute rather than chronic renal problems. Fourth, although few cases of albuminuria were based on 24 hour urine collections, over- or under- collection of urine could have increased or decreased urine albumin values respectively. Fifth, there are limitations to the MDRD eGFR equation particularly at eGFRs = > 60 ml/min/1.73 m^2^[30-32]. Accordingly, we have presented both overall data for those with eGFRs = > 60 ml/min/1.73 m^2^ as well as sub-group information for those with eGFRs = > 90 and 60–90 ml/min. Finally, our cross-sectional design did not allow the longitudinal follow up of people as they transitioned from diabetes diagnosis through the CKD stages. Importantly, this did not permit us to determine mortality rates by diabetes duration, eGFR and presence of albuminuria. The latter information would have allowed us to further clarify the impact of differential mortality related to the competing risks of ESRD and death. In addition, the cross-sectional design prevents us from making causal inferences and the use of the terms “determinant” and “co-morbidity” is not intended to imply that our findings have established a cause-effect relationship.

## Conclusions

Our results demonstrate that, despite the role of genetic [33,34] and pre-natal factors [35] in the development of diabetic kidney disease among Indigenous North Americans, the development of albuminuria was more immediately related to quality of diabetes care and other modifiable risk factors such as smoking. Failure to achieve or even approach CPG targets for glycemic, systolic blood pressure and possibly cholesterol control clearly discriminated between those with and without albuminuria and was related to obvious deficiencies in pharmacologic and lifestyle interventions. Why a small proportion of those with early diabetic nephropathy progressed to eGFRs <60 ml/min/1.73 m^2^ also appeared related to quality of diabetes care, but increasing age and duration of diabetes also emerged as dominant factors. Of particular importance in explaining the development of diabetic ESRD, those with this devastating complication were almost 20 years younger at age of diabetes diagnosis than those with Stage 3 CKD. The most plausible explanation for this observation was an age-related survival benefit that allowed those with an earlier age of diabetes onset to live long enough to develop ESRD. This requires confirmation using longitudinal studies that analyze the competing risks of ESRD and death from the age of diabetes diagnosis. Nonetheless, the overall message is clear. Rate disparities in diabetic ESRD and earlier CKD Stages both within FN populations and between FN and others are potentially remediable through early diagnosis of diabetes, smoking cessation and by meeting established CPG targets for glycemic, blood pressure and cholesterol control. Finally, this study once again highlights the need for communities and health care systems to promote healthy lifestyles and develop effective primary prevention initiatives [36,37]. It is possible that even delaying the onset of diabetes among FN could substantially reduce the incidence of ESRD.

## Competing interests

The authors declare that they have no competing interests that are relevant to this study.

## Authors’ contributions

RD researched data, contributed to the discussion and wrote the manuscript. MN researched data, contributed to the discussion and reviewed/edited the manuscript. SH researched data, contributed to discussion and reviewed/edited the manuscript.

## Pre-publication history

The pre-publication history for this paper can be accessed here:

http://www.biomedcentral.com/1471-2369/13/57/prepub

## References

[B1] Burrows NR Narva AS Geiss LS Engelgau MM Acton KJ End-stage renal disease due to diabetes among southwestern American Indians, 1990–2001 Diabetes Care 2005 28 5 1041 1044 10.2337/diacare.28.5.1041 15855564

[B2] Naqshbandi M Harris SB Esler JG Antwi-Nsiah F Global complication rates of type 2 diabetes in Indigenous peoples: A comprehensive review Diabetes Res Clin Pract 2008 82 1 17 10.1016/j.diabres.2008.07.017 18768236

[B3] Dyck RF Osgood ND Lin TH Gao A Stang MR End-stage renal disease in people with diabetes: A comparison of First Nations people and other Saskatchewan residents from 1981–2005 Can J Diab 2010 34 4 324 333

[B4] Dyck RF Osgood N Lin TH Gao A Stang MR Epidemiology of diabetes mellitus among First Nations and non-First Nations adults Can Med Assoc J 2010 182 3 249 256 10.1503/cmaj.090846 20083562 PMC2826466

[B5] Hanley AJG Harris SB Mamakeesick M Goodwin K Fiddler E Hegele RA Spence JD House AA Brown ED Schoales B McLaughlin JR Klein R Zinman B Complications of type 2 diabetes among Aboriginal Canadians Diabetes Care 2005 28 8 2054 2057 10.2337/diacare.28.8.2054 16043760

[B6] Dyck RF Sidhu N Klomp H Cascagnette PJ Teare GF Differences in Glycemic Control and Survival Predict Higher ESRD Rates in Diabetic First Nations Adults Clin Invest Med 2010 33 6 E390 E397 21134341 10.25011/cim.v33i6.14590

[B7] Maple-Brown LJ Cunningham J Hodge AM Weeramanthri T Dunbar T Lawton PD Zimmet PZ Chadban SJ Polkinghorne KR Shaw JE O'Dea K High rates of albuminuria but not of low eGFR in urban indigenous Australians: the DRUID study BMC Publ Health 2011 19 11 346 10.1186/1471-2458-11-346 PMC3112138 21595912

[B8] Gao S Manns BJ Culleton BF Tonelli M Quan H Crowshoe L Ghali WA Svenson LW Hemmelgarn BR for the Alberta Kidney Disease Network: Prevalence of chronic kidney disease and survival among Aboriginal people J Am Soc Nephrol 2007 18 2953 2959 10.1681/ASN.2007030360 17942955

[B9] Pavkov ME Bennett PH Knowler WC Krakov J Seivers ML Nelson RG Effect of youth-onset type 2 diabetes mellitus on end-stage renal disease and mortality in young and middle-aged Pima Indians JAMA 2010 296 4 421 426 16868300 10.1001/jama.296.4.421

[B10] Dyck RF Tan L Rates and outcomes of diabetic end-stage renal disease among registered native people in Saskatchewan Can Med Assoc J 1994 150 203 208 8287342 PMC1486186

[B11] Canadian Institute for Health information 2007 Annual Report – Treatment of end stage organ failure in Canada, 1996 to 2005 2008 Ottawa, CIHI

[B12] Harris SB Naqshbandi M Bhattacharyya O Hanley AJG Zinman B CIRCLE Study Group Major gaps in diabetes clinical care among Canada’s First Nations: Results of the CIRCLE Study Diabetes Res Clin Pract 2011 92 272 279 10.1016/j.diabres.2011.02.006 21376415

[B13] First Nations Centre OCAP: Ownership, control, access and possession 2007 National Aboriginal Health Organization, Ottawa, ON http://www.naho.ca/firstnations/english/ documents/FNC-OCAP_001.pdf (originally accessed October 28, 2010

[B14] Canadian Diabetes Association Clinical Practice Guidelines Expert Committee Canadian Diabetes Association 2008 clinical practice guidelines for the prevention and management of diabetes in Canada. Can J Diabetes 2008 32 Supplement 1 S1 S201 10.1016/j.jcjd.2013.01.009 24070926

[B15] Levey AS Bosch JP Lewis JB Greene T Rogers N Roth D A more accurate method to estimate glomerular filtration rate from serum creatinine: a new prediction equation Ann Intern Med 1999 130 461 70 10075613 10.7326/0003-4819-130-6-199903160-00002

[B16] Nelson RG Greene T Beck GJ Estimating GFR by the MDRD and Cockcroft-Gault equations in Pima Indians (abstract) J Am Soc Nephrol 2003 14 134a

[B17] National Kidney Foundation K/DOQI clinical practice guidelines for chronic kidney disease: evaluation, classification, and stratification Am J Kidney Dis 2002 39 Suppl 1 S1 S266 11904577

[B18] Jolly SE Mete M Wang H Zhu JH Ebbesson SOE Voruganti S Comuzzie AG Howard BV Umans JG Uric acid, hypertension, and chronic kidney disease among Alaska Eskimos: the genetics of coronary artery disease in Alaska Natives (GOCADAN) Study J Clin Hypertens 2012 14 2 71 77 10.1111/j.1751-7176.2011.00574.x PMC3507473 22277138

[B19] Whaley-Connell AT Sowers JR McFarlane SI Norris KC Chen SC Li S Qiu Y Wang C Stevens LA Vassalotti JA Collins AJ Kidney Early Evaluation Program Investigators Diabetes mellitus in CKD: Kidney Early Evaluation Program (KEEP) and National Health and Nutrition and Examination Survey (NHANES) 1999–2004 Am J Kidney Dis 2008 51 (4)Suppl 2 S21 S29 18359404 10.1053/j.ajkd.2007.12.013

[B20] Shara NM Wang H Valaitis E Pehlivanova M Carter EA Resnick HE Wang W Umans JG Lee ET Howard BV Devereux RB Wilson PWF Comparison of Estimated Glomerular Filtration Rates and Albuminuria in Predicting Risk of Coronary Heart Disease in a Population with High Prevalence of Diabetes Mellitus and Renal Disease Am J Cardiol 2011 107 3 399 405 10.1016/j.amjcard.2010.09.036 21257005 PMC3035999

[B21] Oster RT Johnson JA Hemmelgarn BR King M Balko SU Svenson LW Crowshoe L Toth EL Recent epidemiologic trends of diabetes mellitus among status Aboriginal adults Can Med Assoc 2011 183 12 E803 E808 10.1503/cmaj.101882 PMC3168663 21788417

[B22] Shurraw S Hemmelgarn B Lin M Majumdar SR Klarenbach S Manns B Bello A James M Chowdhury Turin T Tonelli M for the Alberta Kidney Disease Network: Association between glycemic control and adverse outcomes in people with diabetes mellitus and chronic kidney disease Arch Intern Med 2011 171 21 1920 1927 10.1001/archinternmed.2011.537 22123800

[B23] The Diabetes Control and Complications Trial Research Group The effect of intensive treatment of diabetes on the development and progression of long-term complications in insulin dependent diabetes mellitus N Engl J Med 1993 329 977 986 8366922 10.1056/NEJM199309303291401

[B24] Bash LD Selvin E Steffes M Coresh J Astor BC Poor glycemic control in diabetes and the risk of incident chronic kidney disease even in the absence of albuminuria and retinopathy – Atherosclerosis Risk in Communities (ARIC) Study Arch Intern Med 2008 168 22 2440 2447 10.1001/archinte.168.22.2440 19064828 PMC2766035

[B25] Gaede P Vedel P Larsen N Jensen GVH Parving HH Pedersen O Multifactorial intervention and cardiovascular disease in patients with type 2 diabetes N Engl J Med 2003 348 383 393 10.1056/NEJMoa021778 12556541

[B26] Harris SB Zinman B Hanley A Gittelsohn J Hegele R Connelly PW Shah B Hux JE The impact of diabetes on cardiovascular risk factors and outcomes in a native Canadian population Diabetes Res Clin Pract 2002 55 2 165 173 10.1016/S0168-8227(01)00316-3 11796183

[B27] Sarnak MJ Levey AS Schoolwerth AC Coresh J Culleton B Hamm LL McCullough PA Kasiske BL Kelepouris E Klag MJ Parfrey P Pfeffer M Raij L Spinosa DJ Wilson PW Kidney disease as a risk factor for development of cardiovascular disease: A statement from the American Heart Association councils on Kidney in Cardiovascular Disease, High Blood Pressure Research, Clinical Cardiology, and Epidemiology and Prevention Circulation 2003 108 2154 2169 10.1161/01.CIR.0000095676.90936.80 14581387

[B28] Di Angelantonio E Chowdhury R Sarwar N Aspelund T Danesh J Gudnason V Chronic kidney disease and risk of major cardiovascular disease and non-vascular mortality: prospective population based cohort study BMJ 2010 341 c4986 10.1136/bmj.c4986 20884698 PMC2948649

[B29] Klomp H Dyck RF Sidhu N Cascagnette PJ Teare GF Measuring Quality of Diabetes Care by Linking Health Care System Administrative Databases with Laboratory Data BMC Research Notes 2010 3 233 8 www.biomedcentral.com/1756-0500/3/233 20807443 10.1186/1756-0500-3-233 PMC2940772

[B30] Stevens LA Coresh J Greene T Levey AS Assessing Kidney Function - Measured and Estimated Glomerular Filtration Rate N Engl J Med 2006 354 23 2473 2483 10.1056/NEJMra054415 16760447

[B31] Glassock RJ Winearls CG eGFR: Readjusting its rating Clin J Am Soc Nephrol 2009 4 5 867 869 10.2215/CJN.01830309 19414566

[B32] Hallan SI Orth SR The KDOQI 2002 classification of chronic kidney disease: for whom the bell tolls Nephrol Dial Transplant 2010 10.1093/ndt/gfq370 (advance access July 1, 2010) 20601368

[B33] Dyck RF Bohm CJ Klomp H Increased frequency of HLA A2/DR4 and A2/DR8 haplotypes in young Saskatchewan Aboriginal people with diabetic end stage renal disease Am J Neph 2003 23 3 178 185 10.1159/000070747 12700434

[B34] Imperatore G Knowler WC Nelson RG Hanson RL Genetics of diabetic nephropathy in the Pima Indians Curr Diab Rep 2001 1 3 275 81 10.1007/s11892-001-0046-2 12643210

[B35] Nelson RG Morgenstern H Bennett PH Intrauterine diabetes exposure and the risk of renal disease in diabetic Pima Indians Diabetes 1998 47 1489 1493 10.2337/diabetes.47.9.1489 9726239

[B36] Macaulay AC Paradis G Potvin L Cross EJ Saad-Haddad C McComber A Serge Desrosiers Kirby R Montour LT Lamping DL Leduc N Rivard M The Kahnawake Schools Diabetes Prevention Project: intervention, evaluation and baseline results of a diabetes primary prevention program with a Native community in Canada Prev Med 1997 26 779 90 10.1006/pmed.1997.0241 9388789

[B37] Gittelsohn J Harris SB Whitehead S Wolever TMS Hanley JG Barnie A Kakegamic L Logan A Zinman B Developing diabetes interventions in an Ojibwa-Cree community in northern Ontario: Linking qualitative and quantitative data Chronic Dis Can 1995 16 157 64

